# Supplementary figures and images for: Chromatin accessibility and gene expression during adipocyte differentiation identify context-dependent effects at cardiometabolic GWAS loci
Source: PLoS Genet. 2021 Oct 26;17(10):e1009865. doi: 10.1371/journal.pgen.1009865 (PMC8570510; doi:10.1371/journal.pgen.1009865)

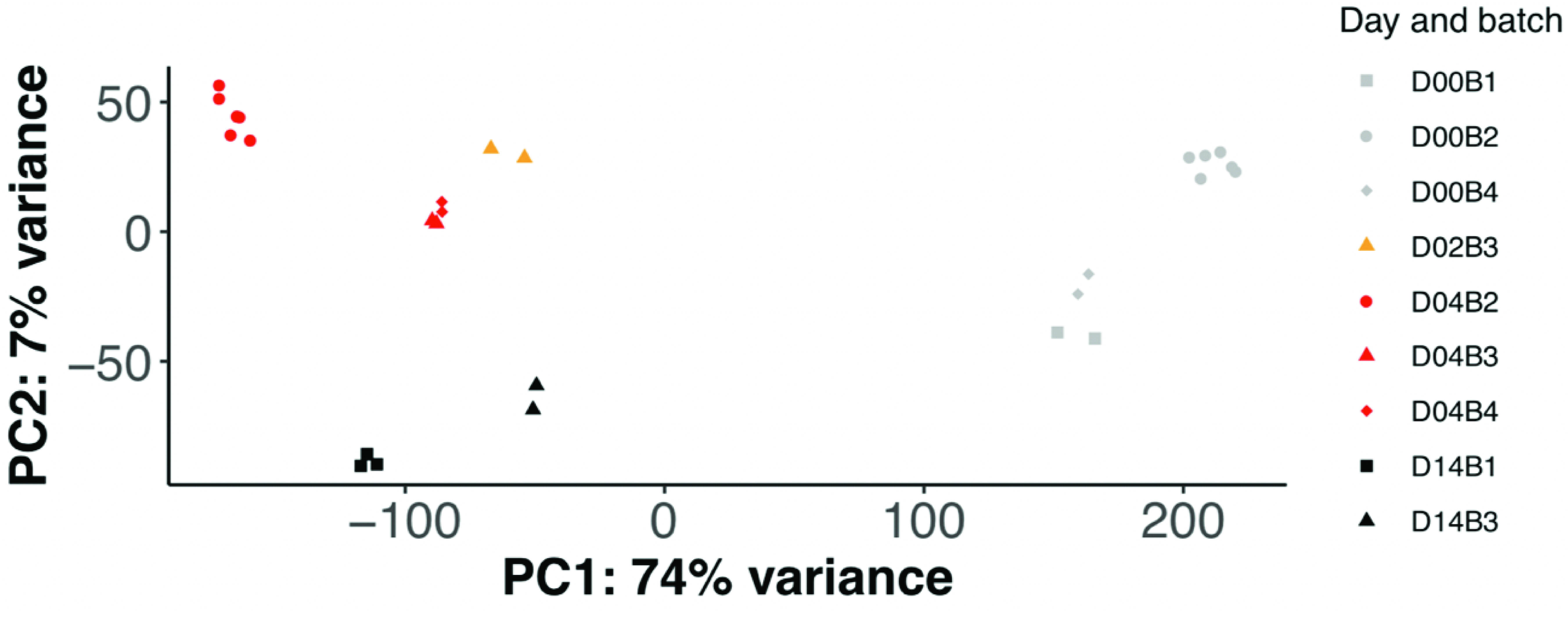

Supplement: S1 Fig — Preadipocytes (D0) are shown in grey, immature adipocytes (D02 and D04) are shown in yellow and red, respectively, and mature adipocytes (D14) are shown in black. Replicates were prepared in four separate batches with batches designated by unique symbols as indicated in the legend. While replicates cluster by batch, PC1 explains 74% of variance and separates preadipocytes from immature and mature adipocytes. Values in S1 Table. (TIF) [file pgen.1009865.s001.tif]

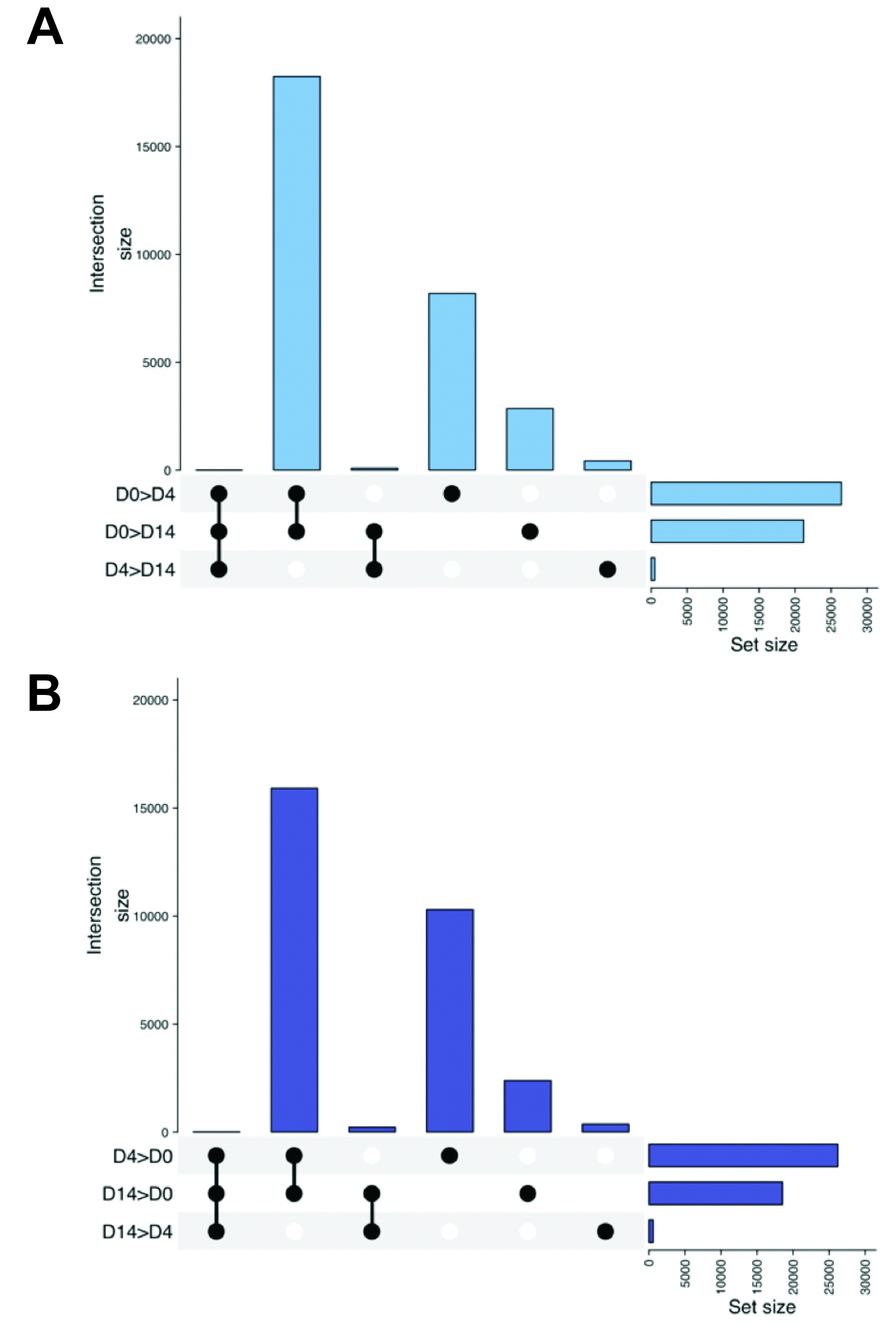

Supplement: S2 Fig — (A-B) Values from S4 Table. (A) Counts of peaks at which earlier timepoints were more accessible than the later timepoints. (B) Counts of peaks at which the later timepoints were more accessible than the earlier timepoints. For both plots, the total number of peaks that show differential accessibility between each pair of timepoints are shown on the bottom right. (TIF) [file pgen.1009865.s002.tif]

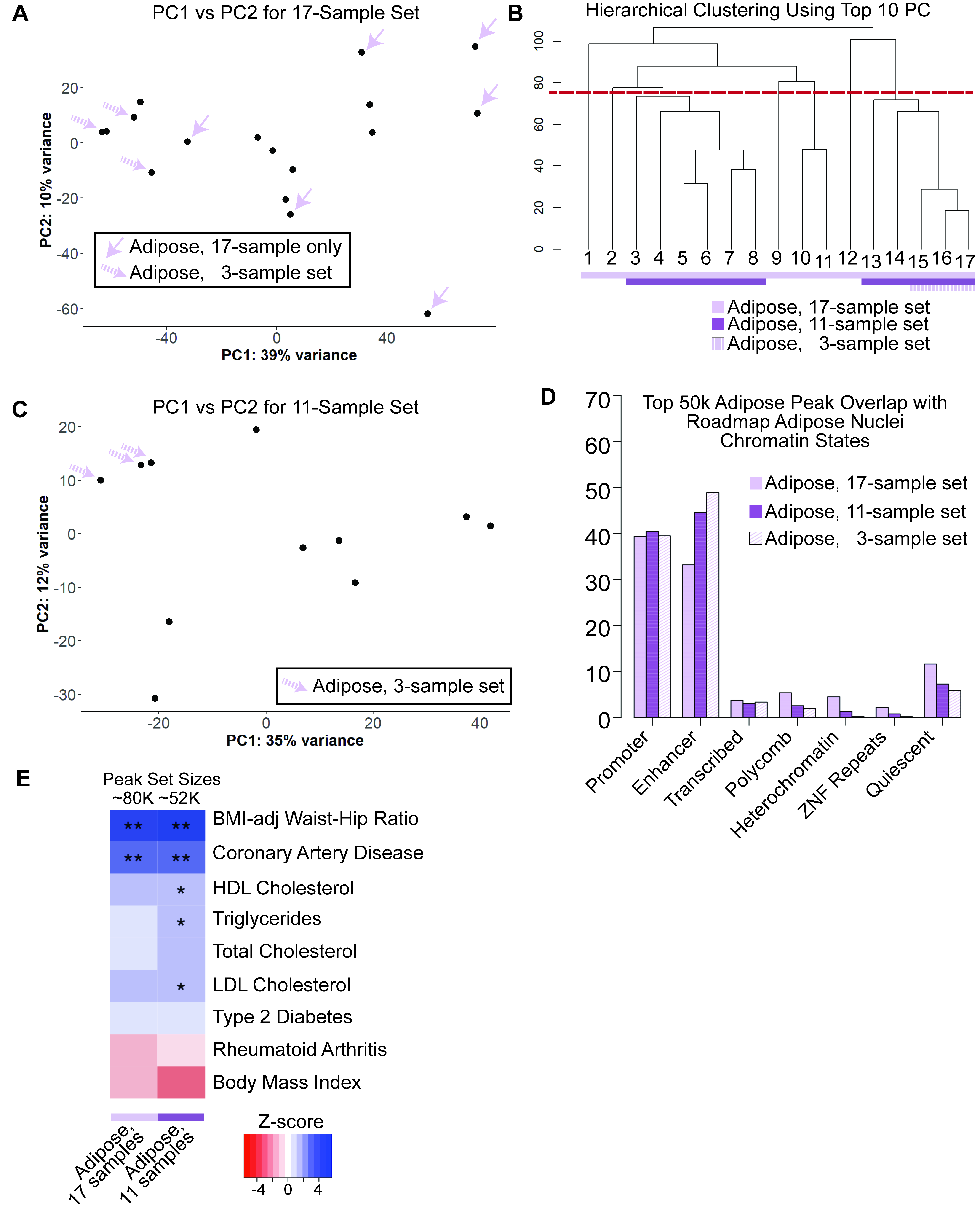

Supplement: S3 Fig — (A) PCA for PC1 (principal component) vs PC2 for all 17 samples that met quality thresholds. Solid light purple arrows indicate samples that are unique to the 17-sample set (excluded from the 11-sample set). Dashed light purple arrows indicate three previously published samples that have been included in the 11- and 17- sample sets. (B) Hierarchical clustering using the top 10 PCs from PCA. The red dashed line indicates the cutoff used to exclude six samples from the 11-sample set. Dark purple indicates samples in the 11-sample set. Dashed light purple indicates samples in the 3-sample set. Sample numbers correspond to library quality metrics in S10 Table. (C) PCA for PC1 vs PC2 for the 11-sample set. Dashed light purple arrows indicate three previously published samples that have been included. (D) Adipose peak overlap with chromatin states of Roadmap adipose nuclei for the three different sample subsets of adipose consensus peaks using the top 50k peaks for each set. Values in S9 Table. (E) Heatmap of cardiometabolic trait GWAS locus enrichment; rheumatoid arthritis was selected for comparison. Peak sets include two sets of adipose tissue peaks. Values in S12 Table. **, P < 0.005; *, P < 0.05. (TIF) [file pgen.1009865.s003.tif]

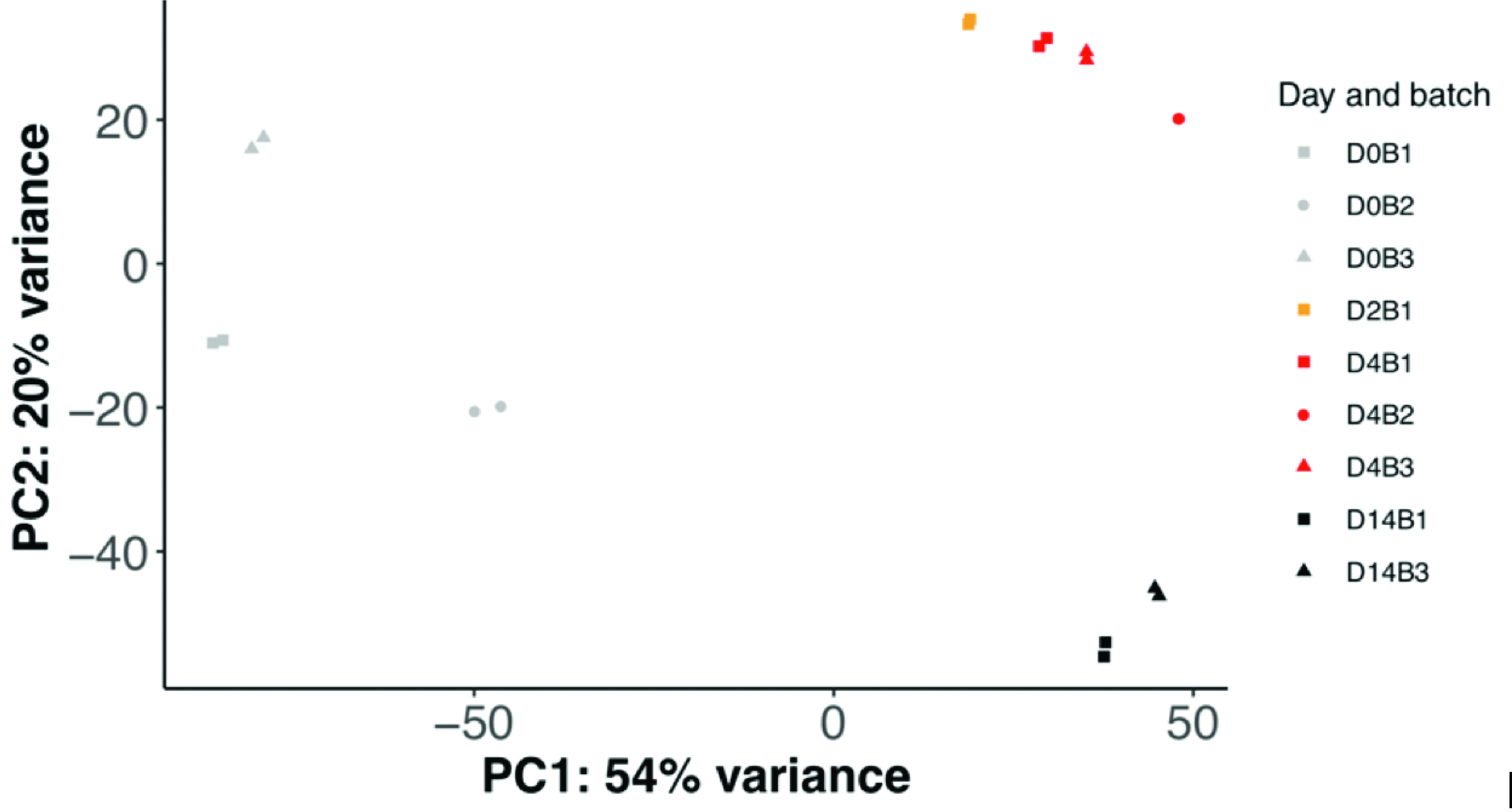

Supplement: S4 Fig — Preadipocytes (D0) are shown in grey, immature adipocytes (D02 and D04) are shown in yellow and red, respectively, and mature adipocytes (D14) are shown in black. Replicates were prepared in three separate batches with batches designated by unique symbols as indicated in the legend. While replicates cluster by batch, PC1 explains 54% of variance and separates preadipocytes from immature and mature adipocytes. Values in S8 Table. (TIF) [file pgen.1009865.s004.tif]

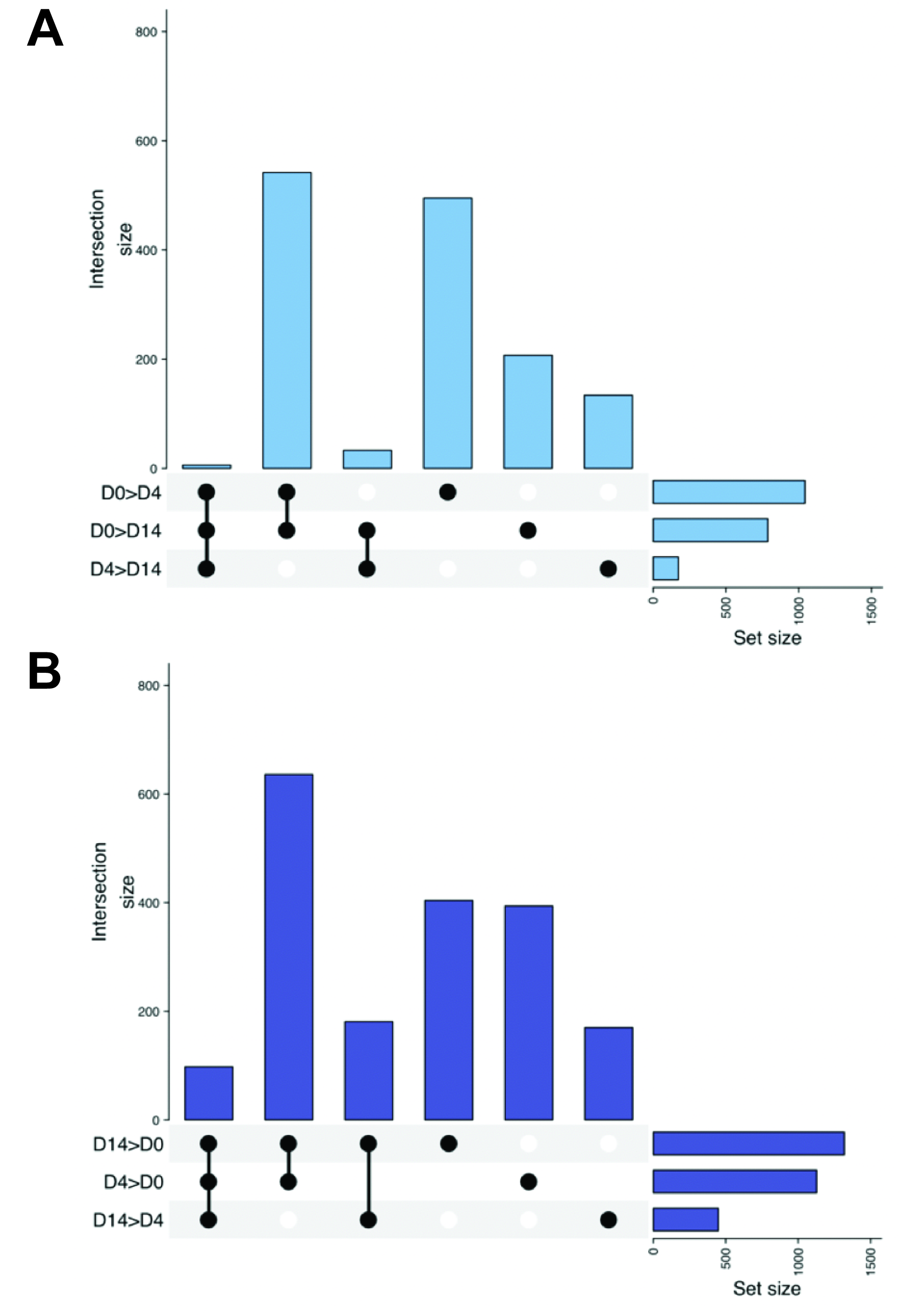

Supplement: S5 Fig — (A-B) Values from S10 Table. (A) Counts of genes that were more highly expressed in an earlier timepoint than a later timepoints. (B) Counts of genes that were more highly expressed in a later timepoints than an earlier timepoints. For both plots, the total number of genes that were differentially expressed between each pair of timepoints are shown on the bottom right. (TIF) [file pgen.1009865.s005.tif]

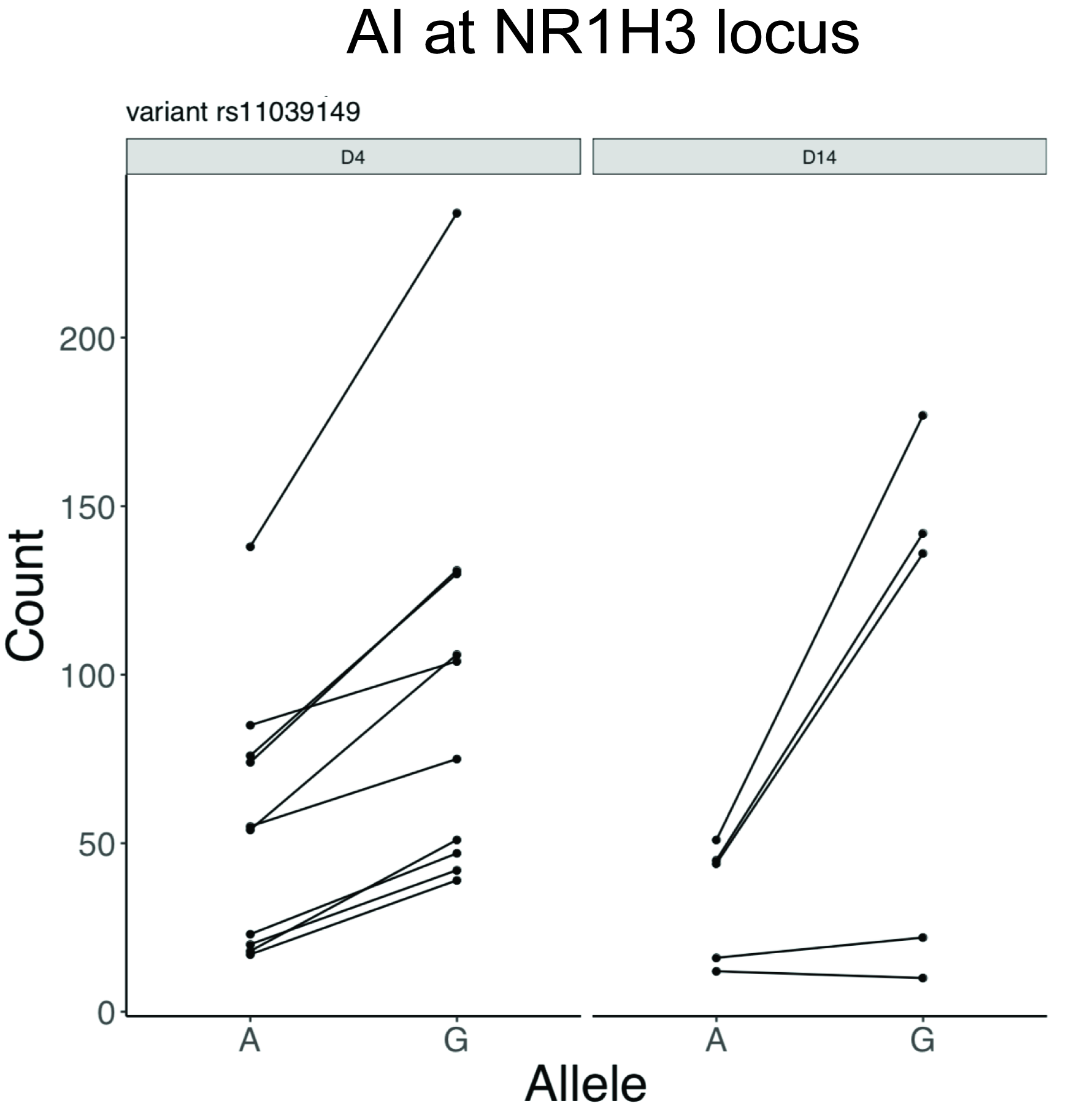

Supplement: S6 Fig — Allele counts of variant rs11039149 within context-dependent peak, peak23801, that is more accessible in D4 compared to D0. This peak was linked to NR1H3 through two methods. Full allelic imbalance results including counts at this variant in S15 Table. (TIF) [file pgen.1009865.s006.tif]

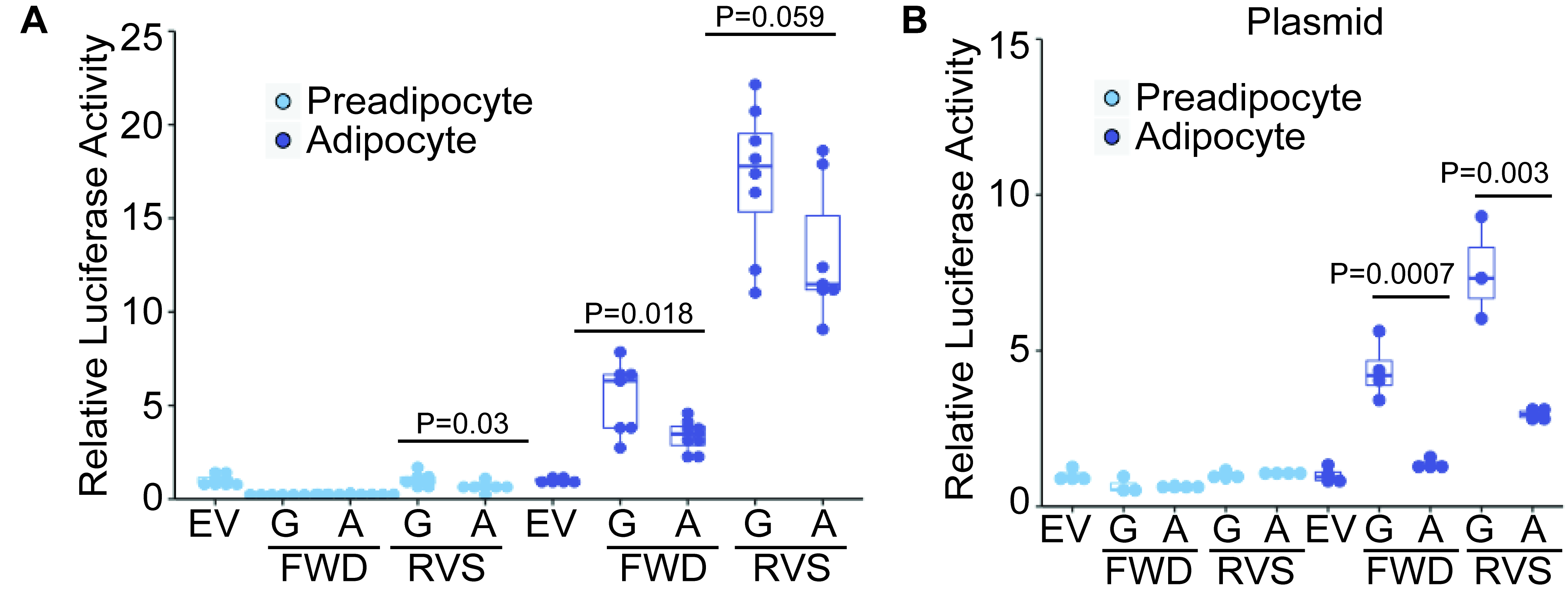

Supplement: S7 Fig — (A-B) Values in S18 Table. (A) Replicate of Fig 4 using the same viral vectors transduced on an independent day. A 592-bp genomic region surrounding peak19405 containing the rs603424-G allele shows increased transcriptional activity compared to the rs603424-A allele in the forward (FWD) and modestly-increased transcriptional activity in the reverse (RVS) orientations in adipocytes (tested at day 7), the context in which chromatin was more accessible compared to preadipocytes. The rs603424-G allele also shows increased transcriptional activity compared to the rs603424-A allele in the RVS orientations in preadipocytes. Dots represent two independent constructs assayed in four replicates each. Luciferase activity was normalized relative to an empty vector (EV). (B) Replicate experiment using plasmid transfections. A 481-bp genomic region surrounding peak19405 containing the rs603424-G allele shows increased transcriptional activity compared to the rs603424-A allele in the forward and reverse orientations in adipocytes (tested at day 3.5), the context in which chromatin was more accessible compared to preadipocytes. Dots represent 5 independent constructs assayed in three replicates each. (TIF) [file pgen.1009865.s007.tif]

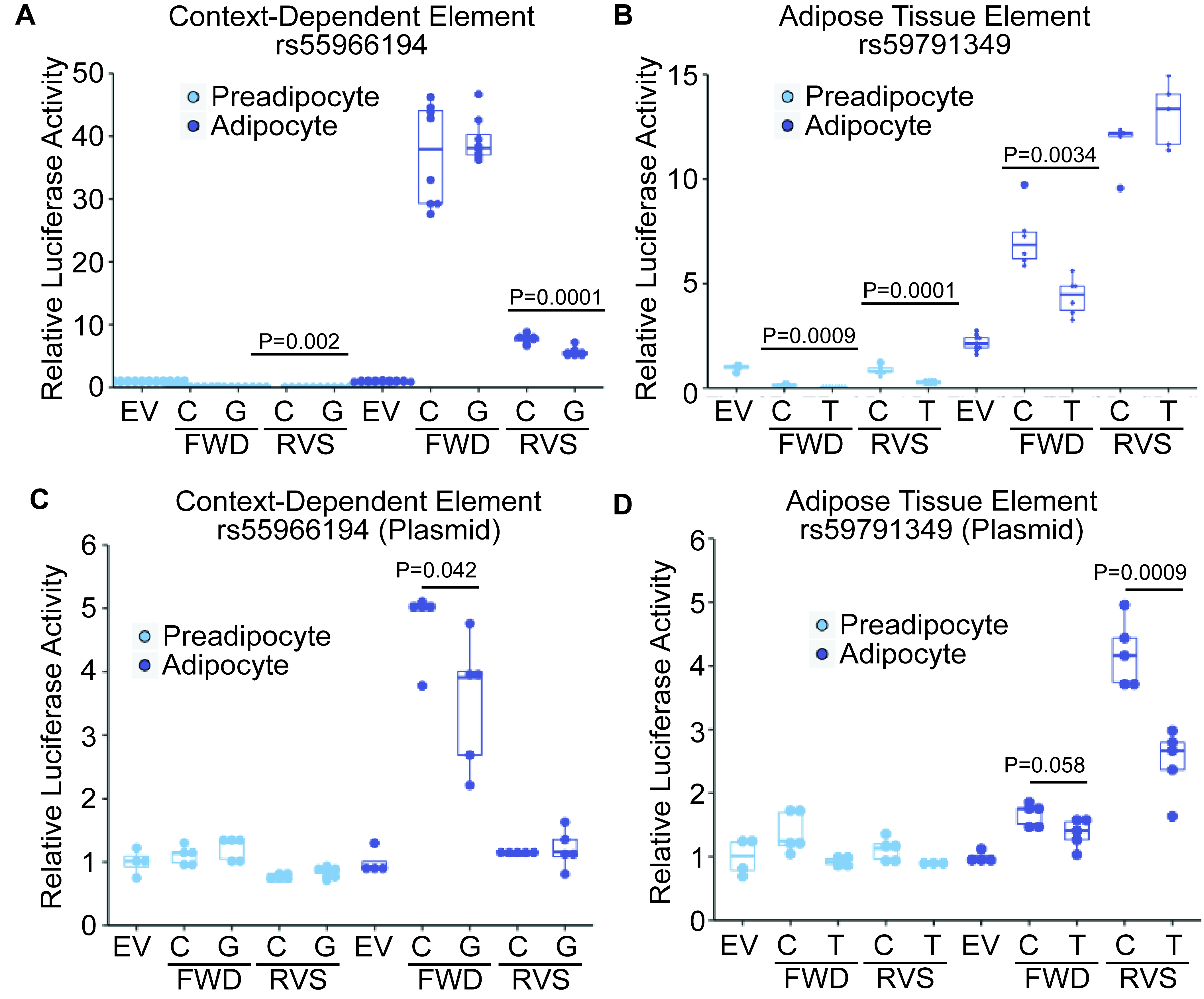

Supplement: S8 Fig — (A-D) Values in S18 Table. (A-B) Replicates of Fig 5 using the same viral vectors transduced on an independent day. (A) A 419-bp genomic region surrounding peak81750 containing the rs555966194-C allele showed increased transcriptional activity compared to the rs555966194-G allele in the reverse (RVS) orientation, but not the forward (FWD), in adipocytes (tested at day 10) and preadipocytes, although transcriptional activity was higher in adipocytes, the context in which chromatin was more accessible compared to preadipocytes. Dots represent two independent constructs assayed from four replicates each. Luciferase activity was normalized relative to an empty vector (EV). (B) A 288-bp genomic region containing the rs59791349-C allele shows increased transcriptional activity compared to the rs59791349-T allele in the reverse orientation and in both preadipocytes and adipocytes (tested at day 9). Dots represent two independent constructs assayed from four replicates each. (C-D) Replicate experiments using plasmid transfections. (C) A 419-bp genomic region surrounding peak81750 containing the rs555966194-C allele shows increased transcriptional activity compared to the rs555966194-G allele in the forward orientation, but not the reverse, in adipocytes (tested at day 3.5). Dots represent 5 independent constructs assayed in three replicates each. (D) A 312-bp genomic region containing the rs59791349-C allele shows increased transcriptional activity compared to the rs59791349-T allele in the forward and reverse orientation and in adipocytes (tested at day 3.5). Dots represent 5 independent constructs assayed in three replicates each. (TIF) [file pgen.1009865.s008.tif]

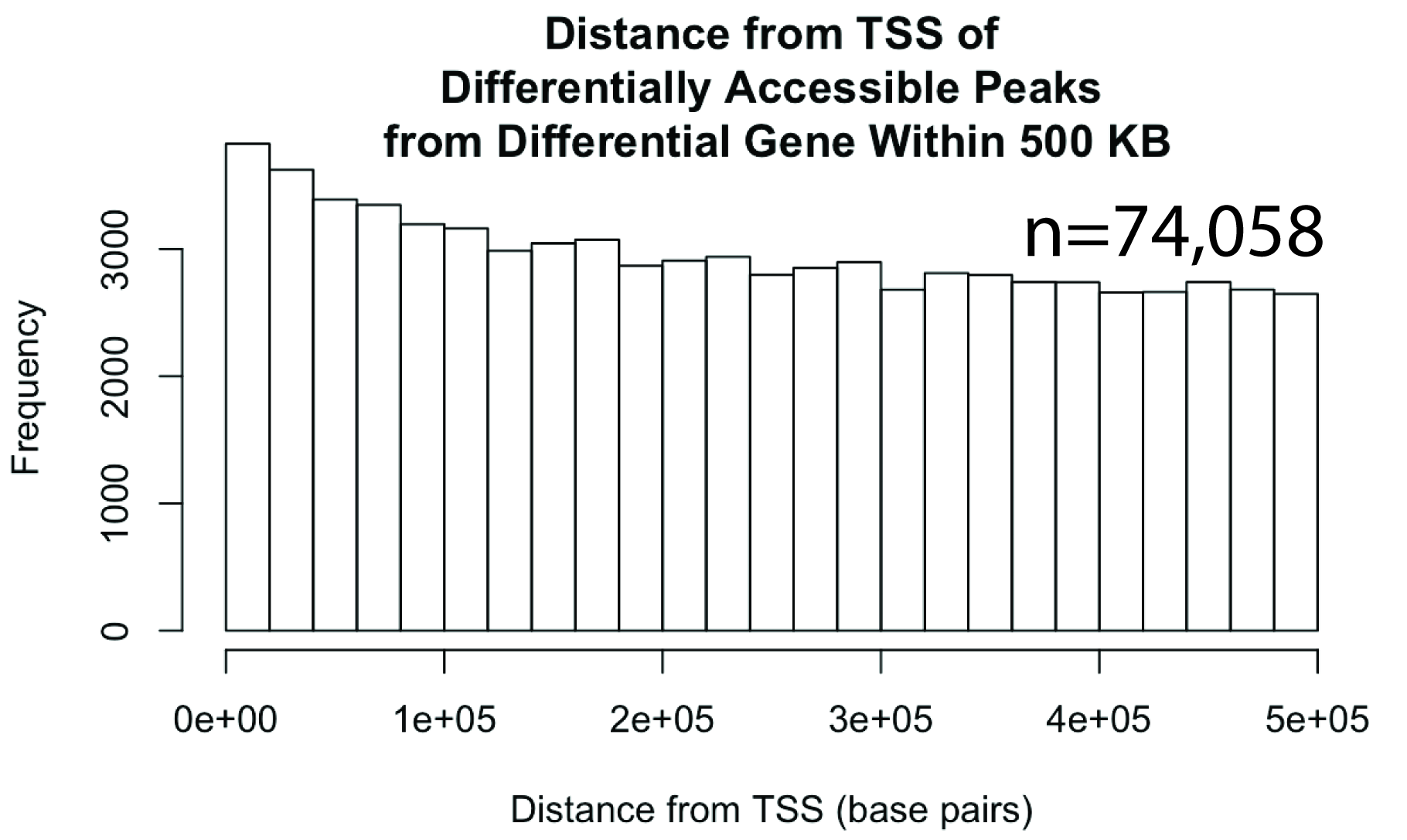

Supplement: S9 Fig — Histogram of distances of differentially accessible peaks to a TSS for all differentially expressed genes within 500,000 bases (n = 74,058). (TIF) [file pgen.1009865.s009.tif]
