# Supplementary material for: Chromatin accessibility and gene expression during adipocyte differentiation identify context-dependent effects at cardiometabolic GWAS loci
Source: PLoS Genet. 2021 Oct 26;17(10):e1009865. doi: 10.1371/journal.pgen.1009865 (PMC8570510; doi:10.1371/journal.pgen.1009865)
Supplement: Attachment — Submitted filename: ResponseToReviewers_PerrinEtAl_Final.docx [file pgen.1009865.s028.docx]

We thank the reviewers for their useful feedback that improved the manuscript. Our response to reviewers is included in **bold** and changes to the text are shown in blue. Data underlying all figures and results are included in supplemental tables with large-scale datasets made available on GEO. A list of editorial changes is provided at the end.

**Reviewer #1:**

In this submission, Perrin et al. aim to identify mechanisms downstream of cardiometabolic GWAS loci through transcriptional and epigenetic profiling of a human cell model of preadipocytes and adipocytes. The relative paucity of eQTLs at GWAS loci may relate partly to the context (cell type, stimulus conditions) of eQTL studies. Like other disease-relevant tissues, adipose tissue is heterogeneous; GWAS variants may therefore act specifically in adipocytes, preadipocytes, or other constituent cells. Using their cell line, the authors identify peaks that are accessible at specific developmental stages, and subsequently assess these for disease relevance.

The authors begin by profiling chromatin accessibility in SGBS cells (Simpson-Golabi-Behmel Syndrome, a preadipocyte cell line) with ATAC-seq at D0 (preadipocyte), D4 (immature adipocyte) and D14 (mature adipocyte) of differentiation. They identify consensus peaks by overlap at each developmental stage. Differentially accessible ("context-dependent") peaks are identified between each pair of timepoints. Most changes in chromatin accessibility appear to occur between day 0 and day 4, then persist to day 14.

ATAC-seq peaks that are more accessible in preadipocytes (D0>D4 and D0>D14) are identified, along with a similar set for adipocytes. The paper defines the adipocyte set as D4>D0 and D14>D0, but the D14>D4 and D14>D0 set would seem to be more specific for mature adipocytes; further motivation for this choice, and examination of its effect on their overall results, would be helpful. Both preadipocyte- and adipocyte-dependent peaks were enriched near genes involved in relevant metabolic processes and appropriate transcription factor motifs, suggesting they capture relevant biology.

**We agree and also expected that peaks more specific to mature adipocytes would be an interesting subset. We identified these ‘mature adipocyte-dependent peaks’ (D14>D0 and D14>D4), but only 233 peaks meet these criteria, suggesting that the major chromatin changes during adipocyte differentiation occur prior to day 4. The 233 peaks specific to mature adipocytes is a very small number to characterize genome-wide changes, which is why we focused primarily on the larger set of 15,919 immature and mature adipocyte-dependent peaks.**

**We added characteristics of these mature adipocyte-dependent peaks to the results text and S5 Table, and additional results can be found on S12 and S15 Tables by examining peaks listed as differentially accessible in D14>D4 and D14>D0. We also explained our motivation to proceed with the larger set of adipocyte-dependent peaks for primary analyses in results, and revised a section of the discussion to make this decision clear.**

**Lines 129-138:** “Most (86%) of the changes in chromatin accessibility between D0 and D14 were observed by D4 (S2 Fig), and only 233 peaks were specifically more accessible in mature adipocytes (D14>D0 and D14>D4; S2 Fig), suggesting that chromatin accessibility changes early after the initiation of differentiation and remains largely stable between D4 and D14. To characterize the major differences, we identified context-dependent peaks more accessible in preadipocytes in both comparisons (D0>D4 and D0>D14; 18,244 peaks, S2 Fig), hereafter called ‘preadipocyte-dependent peaks’, and context-dependent peaks more accessible in immature and mature adipocytes (D4>D0 and D14>D0; 15,919 peaks, S2 Fig), hereafter called ‘adipocyte-dependent peaks’.”

**Lines 146-148:** “Mature adipocyte-dependent peaks were enriched near genes with roles in cardiovascular development (S5 Table).”

**Lines 460-463:** “Similarities between D4 and D14 also led us to focus on the subsets of context-dependent peaks that were specific to preadipocytes (D0>D4 and D0>D14) or adipocytes (D4>D0 and D14>D0), rather than the limited number that were specific to mature adipocytes (D14>D0 and D14>D4).”

In parallel, the authors generate a consensus map of chromatin accessibility in subcutaneous adipose tissue from 11 healthy donors (17 prior to QC) using similar methods. Sequencing details are somewhat heterogeneous across the study, with variable read length and sequencing method (paired-end vs. single-end). This is addressed to an extent in their QC but remains a potential limitation.

**We added text to the discussion to acknowledge the limitations of the heterogenous sequencing protocols.**

**Lines 432-437:** “A technical source of variation between our profiles could be due to heterogenous sequencing protocols with a mix of paired-end, single-end, and variable read lengths. We addressed the heterogenous sequencing protocols in our analyses as described in the methods, but it could contribute to differences between libraries. Reassuringly, our SGBS ATAC libraries cluster by day despite differences in sequencing parameters.”

To link changes in chromatin accessibility to potential gene regulatory changes, the authors use RNA-seq to identify gene expression alterations at each stage of adipocyte differentiation. They link context-dependent peaks with potential gene targets by consensus of 2/3 methods: (a) overlap with promoter-capture Hi-C regions, (b) overlap with adipose eQTL signals, and (c) Hi-C overlap or eQTL overlap with additional context-dependent expression. Each of these methods has significant limitations in isolation. eQTL signals overlapping context-dependent accessibility regions do not necessarily have a context-dependent effect. Hi-C regions are frequently large, and the authors included overlaps as small as a single base pair. A more stringent overlap threshold may more precisely identify candidate peaks of interest.

**There is no standard overlap threshold, therefore we investigated the context-dependent peak fraction overlapping promoter capture Hi-C regions. This allowed retention of single base pair overlaps and assessment of different levels of more stringent overlap. The percent overlap is now reported in S12 Table (formerly S11). We added text to address how stringency affected results.**

**Lines 234-236:** “In the first approach, we identified context-dependent peaks that overlapped adipocyte promoter capture Hi-C regions [33,34] (overlap>=1 base pair, Fig 2A, S12 Table). We identified 14,080 peaks linked to 9,080 genes (28,696 peak-gene pairs).”

**Lines 236-242:** “We investigated the extent to which increasing the overlap threshold between peaks and Hi-C fragments would change our results. Of the 14,594 peak-Hi-C fragment overlapping pairs (some peaks overlap more than one Hi-C fragment and vice versa), 12,380 (85%) have over 50% of peak bases within the Hi-C fragment and 10,329 (71%) have the entire peak within the Hi-C fragment (S12 Table), suggesting that we would obtain similar results using more strict overlap thresholds.”

A total of 5,145 peaks were linked to the same gene by >=2 approaches, with 78 peaks linked to the same gene by all three approaches. The 78 peaks that link to the same gene by all three methods would seem logical candidates for further mechanistic exploration, but the authors focus on the larger set of traits linked by >=2 methods.

**We appreciate the importance of this feedback, also provided by Reviewer 2. We agree that there are interesting logical candidates in the set of 78 peaks linked to a gene by all three approaches, however we did not limit to this set due to the advantages and disadvantages of each approach, which could result in missing interesting candidates. To emphasize the peaks linked by all three methods, we sorted the relevant table (S12, formerly S11) so that peaks linked by all three methods are easier to find, and we added additional characterization of the set linked by all three approaches to the results text.**

**Lines 272-278:** “Of 16,076 total peaks linked to a gene through at least one of the three approaches (Table S12), 78 peaks were linked to the same gene through all three approaches and 5,145 peaks were linked to the same gene through two or more approaches (Fig 2E). Of the 78 peaks linked to 59 genes through all three approaches, interesting candidate regulatory elements include four peaks linked to CDKN2B, whose gene product has known roles in cell cycle control and whose regulation has been linked to coronary artery disease [36,37].”

**Lines 281-284:** “Although peaks linked by all three approaches have the most supporting evidence, to prevent overlooking interesting candidates we considered peaks linked by two or more methods when evaluating candidates for functional evaluation.”

**We further described the decision to include peaks linked by two or more approaches in the discussion here.**

**Lines 492-497:** “Due to the advantages and disadvantages of the different approaches, the largely different peak-to-gene links detected were not surprising. Using multiple approaches to link regulatory elements to candidate genes can overcome the limitations of each approach, and genes identified by multiple methods can increase confidence, although genes linked by even a single method merit further investigation.”

They identify GWAS signals (proxies with r^2>0.8) that overlap context-dependent peaks and link these to gene targets using their consensus method. While a number of these may represent disease mechanisms, these should be viewed as mechanistic candidates. Simple overlap is not sufficient to establish causal mechanistic links between GWAS variants, putative context-dependent regulatory elements within context-dependent chromatin, and downstream genes. Further work will be required to validate hypotheses that arise from this work.

**We added discussion text to acknowledge the limitation of our transcriptional reporter assays and additional experiments that would be needed to validate mechanisms.**

**Lines 541-544:** “Our reporter assays demonstrate allelic differences in transcriptional activity for elements at two loci, however, additional experiments are needed to validate specific regulatory elements within these peaks in the context of chromatin accessibility and the effect on regulation on the predicted gene.”

Supporting the relevance of context-dependent chromatin regions, stratified LD score regression is used to demonstrate heritability enrichment for waist:hip ratio adjusted for BMI in adipocyte-dependent and adipose tissue peaks but not in preadipocyte-dependent peaks. Coronary artery disease was also enriched in adipose tissue peaks and SGBS peaks defined at day 0 and day 4. Allelic imbalance is observed in several peaks, but this analysis was limited by the fact that all SGBS cells have the same genotype.

**We acknowledge the limitation of using genotypes from a single cell line and modified the wording to make the limitation clear.**

**Lines 500-503:** “Although we only tested AI at heterozygous variants from one individual, which limits heterozygous sites available for testing, we identified 55 peaks containing GWAS variants that exhibited AI, 13 of which were linked to genes. ATAC-seq in additional cell lines with diverse genotypes would improve ability to detect AI.”

**We also acknowledge the advantages of using multiple cell lines.**

**Lines 506-509:** “Our results demonstrate that AI in ATAC-seq reads from one individual can be used to predict regulatory variants, although identifying AI in larger sample sizes would lead to more comprehensive and robust results and more genetic variants.”

Data from this study will be a useful resource for future studies of GWAS mechanism within adipose tissue, but at this point specific hypotheses are not validated. Demonstration of context-dependent chromatin accessibility is an important first step, but the present study cannot identify specific regulatory elements that are contained within these peaks.

**We added text to acknowledge the limitation of our transcriptional reporter assays and additional experiments that would be needed to validate mechanisms.**

**Lines 541-544:** “Our reporter assays demonstrate allelic differences in transcriptional activity for elements at two loci, however, additional experiments are needed to validate specific regulatory elements within these peaks in the context of chromatin accessibility and the effect on regulation on the predicted gene.”

The SGBS cell line offers an elegant approach to dissociate preadipocyte and adipocyte biology, but disease relevance may be somewhat limited by the artificial conditions in which the cell line is grown.

**We added text to acknowledge the limitation of the SGBS cell line.**

**Lines 437-442:** “Additionally, while SGBS cells are a useful human adipocyte model, some aspects of the chromatin accessibility profile could be due to the cells growing in culture or the overgrowth syndrome disease state that allows the cells to grow without being transformed. To address these limitations, we identified SGBS peaks that overlapped adipose tissue peaks, including the peak we tested at the SCD locus.”

**Reviewer #2:**

Perrin et al present a nice manuscript regarding ATAC seq and RNA seq in SGBS cells, day 4 and 14 compared to ATAC seq from 11 SAT human samples (METSIM). Found > 50 K context dependent chromatic accessibility in all timepoint comparisons. Linked context dependent peaks based on Hi-C data overlap with eQTL and gene expression. Found GWAS for cardiometabolic traits, adipocyte dependent peaks enriched for WHR.

Overall the goals are good: Find out how GWAS loci work, Map regulatory elements with Chromatic accessibility (ATAC Seq) and in an appropriate cell/tissue (Adipose tissue important in CM traits, lots of eQTLs in adipose). This group has a track record in this area and the methods seem sound.

I think a few things will improve the paper.

-16K context dependent peaks linked to a gene -> 5K linked to 1.6K genes (Fig 2 E) -> 78 linked to same gene through all 3 approaches...I wonder what are these 78 genes? Seems like these are low hanging fruit as it were?

**We appreciate the importance of this feedback, also provided by Reviewer 1. We agree that there are interesting logical candidates in the set of 78 peaks linked to a gene by all three approaches, however we did not limit to this set due to the advantages and disadvantages of each approach which could result in missing interesting candidates. To emphasize the peaks linked by all three methods, we sorted the relevant table (S12, formerly S11) so that peaks linked by all three methods are easier to find, and we added additional characterization of the set linked by all three approaches.**

**Lines 272-278:** “Of 16,076 total peaks linked to a gene through at least one of the three approaches (Table S12), 78 peaks were linked to the same gene through all three approaches and 5,145 peaks were linked to the same gene through two or more approaches (Fig 2E). Of the 78 peaks linked to 59 genes through all three approaches, interesting candidate regulatory elements include four peaks linked to *CDKN2B*, whose gene product has known roles in cell cycle control and whose regulation has been linked to coronary artery disease [35,36].”

**Lines 281-284:** “Although peaks linked by all three approaches have the most supporting evidence, to prevent overlooking interesting candidates we considered peaks linked by two or more methods when evaluating candidates for functional evaluation.”

**We further described the decision to include peaks linked by two or more approaches in the discussion here.**

**Lines 492-497:** “Due to the advantages and disadvantages of the different approaches, the largely different peak-to-gene links detected were not surprising. Using multiple approaches to link regulatory elements to candidate genes can overcome the limitations of each approach, and genes identified by multiple methods can increase confidence, although genes linked by even a single method merit further investigation.”

-p.10…Applaud the use of many different ways to try to link peaks to genes…But seems like this is necessarily limited by the availability of Hi-C data from preadipocytes. Was this not looking “under the lamp post where there is light”? Similar to having eQTL data from adipocytes but not pre-adipocytes. I think even more focus could potentially be given to adipocytes given this limitation.

**We revised acknowledgements of the limitations of using adipocyte Hi-C data and adipose eQTL data to clarify the limitations and our primary focus on adipocyte-dependent peaks.**

**Lines 479-482:** “Although adipose tissue is heterogenous, it is known to contain more adipocytes than preadipocytes [16,31]. Therefore, the eQTL method also could have detected connections for the D4 and D14 timepoints better than for D0.”

**Lines 512-513:** “Due to the bias towards adipocytes of our methods to link peaks to genes, we focused on regulatory elements more accessible in adipocytes.”

**We further acknowledged the limitation of using only adipocyte Hi-C data here.**

**Lines 469-472:** “However, physical proximity does not necessarily imply a regulatory relationship, and the data we used was for adipocytes, not preadipocytes, and therefore could have detected connections for the D4 and D14 timepoints better than for D0.”

-Nice to see the heritability enrichment (p.12 and Table S13). The striking findings with WHR are important as are the findings related to HDL, TG, and CAD which all point to a “metabolic syndrome” overlap. Overall the data presented on table S13 should probably emphasize normalized count overlapping adipose dependent peaks rather than any context dependent peak. At least when I order the table in this regard it makes a lot of biological sense. Associations with WHR, HDL, birth weight, TG, CVD, bone density Afib are all expected. However, other interesting biological insight is that breast cancer is in the top 25 as are eczema, and many pulmonary traits (FEV1). This is quite interesting. Would be interested to see if genetically programmed WHR is causally related to FEV1 as a later paper. Also the relationship to intraocular pressure is not immediately intuitive though I am not an eye or glaucoma expert.

**We appreciate the feedback to emphasize the adipocyte-dependent peaks and did so by resorting Table S14 (previously S13). In Figure 3B, we also highlight the top terms for adipocyte-dependent peaks first for emphasis. We also agree that investigating links between WHR and other less expected traits would be an interesting route for a later paper. Metabolic syndrome has been linked to both lung function and intraocular pressure, so we added references to these established links.**

**Lines 315-323:** “The most enriched terms for adipocytes included waist-hip ratio, cholesterol, inflammatory traits, and birthweight, whereas the most enriched terms for preadipocytes included atrial fibrillation and inflammatory traits. We also observed enrichments for traits with less apparent, but established connections to cardiometabolic traits, including forced expiratory volume, a measure of lung function that has been shown to be lower in individuals with metabolic syndrome and high body fat percentage [41,42], and intraocular pressure, which has been shown to be higher in individuals with metabolic syndrome and markers of obesity [43,44].”

That table also seems a bit strange to me in one way. There are about 290 traits that have the same number of “total count of signals from catalog, n=1277 with same number of “count overlapping any context dependent peak, n=233) giving identical “normalized count overlapping…, n=18.25). Why is this?

**We appreciate that the reviewer found this phenomenon, which reflects how the GWAS catalog had been curated, which we verified with the curators. We addressed this unusual aspect of curation as described in more detail below. In our updated analysis, most of the top terms remained unchanged and we updated S14 table (formerly S13) and Figure 3B to reflect this change. One more noticeable change is the addition of C-Reactive Protein, a relevant metabolic trait, to top terms for adipocyte-dependent peaks.**

**In the GWAS catalog curation, the GWAS disease trait was listed as "Blood protein levels" and the associated EFO terms were 329 separate blood proteins, such as "SPARC-like protein 1 measurement" and "small nuclear ribonucleoprotein F measurement". Therefore, it appeared that the GWAS disease trait was switched with the EFO terms in the GWAS catalog. We received confirmation from the GWAS catalog curators that for some traits, including "Blood protein levels", a more general trait was listed in the "DISEASE/TRAIT" column and the more specific trait was listed in a secondary "MAPPED_TRAIT" column, and the more general trait was listed as the specific GWAS trait in the file linking disease traits to EFO terms. After further investigation, we found many more cases where one GWAS disease trait was linked to many different EFO terms, suggesting that this phenomenon was not limited to "Blood protein levels". Because our goal in using EFO terms was to reduce the complexity of the traits in the GWAS catalog, we decided to remove any GWAS trait that mapped to 5 or more EFO terms for the enrichment analysis presented in S14 table (formerly S13) and Figure 3B. This filter only removed less than 1% of the traits in the GWAS catalog. We allowed a trait to map to up to 4 EFO terms because it is expected that some traits can map to multiple, related EFO terms. We chose 5 as the threshold based on visual inspection of a histogram listing the number of EFO terms mapped to each trait. However, we retained these GWAS traits and EFO terms in S15 table for completeness.**

**We added text to the methods to describe our updated analysis.**

**Lines 817-821:** “Because our goal in using EFO terms is to reduce the complexity of the GWAS catalog traits, we removed any GWAS traits that mapped to 5 or more EFO terms for our analyses that count EFO terms, which only removed <1% of GWAS traits. However, we retained all GWAS traits and EFO terms in S15 Table for reference.”

I guess I would like to see a bit more of the discussion focused on medical relevance and the results less about presenting "numbers" of peaks with overlap (which could be gleaned from the figures)...More about why all this work is medically relevant would help the reader.

**We added text to address medical relevance of these results.**

**Lines 315-325:** “The most enriched terms for adipocytes included waist-hip ratio, cholesterol, inflammatory traits, and birthweight, whereas the most enriched terms for preadipocytes included atrial fibrillation and inflammatory traits. We also observed enrichments for traits with less apparent, but established connections to cardiometabolic traits, including forced expiratory volume, a measure of lung function that has been shown to be lower in individuals with metabolic syndrome and high body fat percentage [41,42], and intraocular pressure, which has been shown to be higher in individuals with metabolic syndrome and markers of obesity [43,44]. Taken together, we found that peaks in adipocytes contribute more to heritability of WHRadjBMI, whereas preadipocytes may contribute more to heritability of CAD, though to a lesser degree.”

**Lines 569-572:** “Additional study of these regulatory elements could lead to a better understanding of the role of adipocytes and adipocyte differentiation in cardiometabolic disease traits as well as other relevant traits we identified through enrichment analyses such as lung function.”

Minor
-I understand looking at enrichment of transcription factor motifs (p.6-7) in ATAC seq peaks…But less so the pathway analyses. Pathway analyses focused on the “nearest” gene (Table S5) will often be misleading as often times the “nearest” gene is not relevant (as elegantly shown in this and other papers).

**We added text to acknowledge the limitation of the pathway analysis:**

**Lines 155-159:** “Thus, adipocyte- and preadipocyte-dependent peaks are found near genes and contain transcription factor motifs relevant to their cell contexts, increasing confidence that these peaks capture relevant biology. Although genomic proximity between regulatory elements and genes is a strong predictor of a regulatory relationship [29], regulatory elements may not always affect the nearest genes.”

-"Context dependent" is a reasonable phrase but non-specific. Could “differentiation-specific” be used?

**We appreciate that differentiation-specific is more specific but we think it could be interpreted as less representative of preadipocyte-dependent peaks also described here. While we focus more on adipocyte-dependent peaks throughout the paper, we prefer the more general context-dependent unless strongly advised otherwise.**

-Seems like there has been a similar ATAC Seq study in more commonly used 3T3-L1 cells (Discussion) which may be mentioned in the Intro. What else was the overlap in findings from the mouse to the human cell line? We all want to understand if the mouse cell line can be used as it is a bit easier than SGBS (which have a limited supply).

**Direct comparison between the 3T3-L1 study and our SGBS study is complicated due to between species analysis and the use of DHS profiles used in the 3T3-L1 study. We added text to the introduction and discussion to address a general comparison.**

**Lines 91-94:** “Chromatin accessibility during adipogenesis has been studied in models such as mouse 3T3-L1 cells [20], however additional studies in human models could improve interpretation of human non-coding genetic variants.”

**Lines 442-445:** “Although it remains challenging to compare between species, we observed enrichment of motifs for well-known adipogenesis transcription factors CEBP, PPAR, and RXR within adipocyte-dependent peaks, consistent with a study of changes during adipogenesis in a 3T3-L1 mouse line [20].”

-Fig 1a indicates ATAC seq in 17 people but abstract says 11 people were used, I see in the Figure legend a subset of 11 was used but what happened to the other 6 (33% of the samples)? p.7 indicates they were outliers due to PCA. But why even bother mentioning these or including the data. You could simply omit all references to the 6 samples from all analyses by saying they did not meet QC or other standards.

**We changed the reference in Figure 1A from 17 to 11 to emphasize that the final set used for consensus adipose peaks was from 11 samples. We retained other references to the 17-sample set because these comparisons justify our choices to remove the six samples that met original QC standards but were eliminated through PC analyses.**

-Fig 1b…order is D0, D14, D4 and 1c is D0, D4, D14…why the difference? Shouldn’t order be temporal?

**We appreciate this feedback and changed figure 1B to temporal order.**

Editorial changes:

We reviewed the manuscript to ensure data underlying all figures and results is represented in tables. To provide values underlying figures, we added S8 and S18 Tables. The addition of S8 Table shifted the numbering of subsequent tables and references were adjusted throughout the text accordingly. We added clear references between figures and tables to aid identifying links between them.

After supplemental table updates in response to reviewer feedback, we recalculated counts for peaks linked to genes and GWAS variants and made minor changes. For example, we now report that we identify 659 peaks linked to a gene by two or more approaches and a GWAS variant, instead of the previously counted 666.
